# EXPANDING RELATIONAL BOUNDARIES OF DEMENTIA CARE

**DOI:** 10.1093/geroni/igz038.671

**Published:** 2019-11-08

**Authors:** Karen A Roberto, J Tina Savla

**Affiliations:** Virginia Tech, Blacksburg, Virginia, United States

## Abstract

Rapid changes in family structures have expanded caregiving boundaries beyond the level of lineal kin to include extended and fictive kin. Guided by stress process and health behavior models, we analyzed semi-structured interviews with 120 family caregivers of persons with dementia (PWD) in rural Appalachia to explore personal/family circumstances that influence the responsibilities nonlineal kin assumed to meet the needs of PwD. Compared to spouse and adult children caregivers, nonlineal caregivers reported that PWD had similar behavioral problems, but greater ADL limitations. They also expressed greater burden, overload and role captivity; yet, they reported higher personal mastery, and perseverance. Although sisters and nieces did not report using any paid services to care for PwD, grandchildren and fictive kin used paid services such as meal delivery, personal care, and respite services. Findings provide new insights into a more elaborated conception of caregiving that considers the transformations occurring in family life today.

